# From the perspective of the Construal Level Theory: Examining the effect of psychological distance on system justification

**DOI:** 10.1111/bjso.70047

**Published:** 2026-01-29

**Authors:** Federica Scarci, Matteo Bonora, Valeria De Cristofaro, Valerio Pellegrini, Mauro Giacomantonio

**Affiliations:** ^1^ Department of Psychology of Developmental and Socialization Processes Sapienza University of Rome Rome Italy; ^2^ Present address: Roma Tre University Rome Italy; ^3^ Present address: University of Campania ‘Luigi Vanvitelli’ Caserta Italy

**Keywords:** construal level, inequality, psychological distance, system justification

## Abstract

The current research examines the relationship between psychological distance and system justification through the lens of the Construal Level Theory. In three experimental studies, we investigated whether and how psychological distance shapes the salience of different levels of social identity relevant to system‐justifying tendencies. In Study 1, we investigated the moderating effect of psychological distance on the relationship between membership in different gender‐based groups and system justification in the context of gender inequality. In Study 2, we investigated the influence of psychological distance on the extent to which individuals with opposing political ideologies justify the system. Finally, Study 3 deepened Studies 1–2 by comparing the impact of lower‐ vs. higher‐level identity threats as a function of psychological distance. Results suggest that psychological distance reduces system justification among typically high‐justifying groups, leading to greater convergence across status and ideological divides. Implications, limitations and future directions are discussed.

## INTRODUCTION

In recent years, intergroup inequality has increased throughout the industrialized world despite numerous efforts to reduce it, and global wealth has accumulated and consolidated in the hands of only a few (Solt, 2020). Group‐based disparities such as ethnicity‐related income disparities and racial biases continue to permeate societies (Richeson & Sommers, 2016), and despite progress, gender pay gaps persist (Eagly & Carli, 2007; Moss‐Racusin et al., 2012).

Numerous psychological mechanisms at the individual, group and system levels have been identified as contributing to the legitimization and maintenance of inequality, both among disadvantaged and privileged group members. A significant contributing mechanism is specific to the system justification motivation, which refers to the motivational tendency to perceive the extant (unequal) status quo as fair, legitimate and desirable (Jost & Banaji, 1994). There is abundant evidence demonstrating the role of system justification in maintaining inequality (Jost et al., 2004) and opposing social change (Osborne et al., 2018). Therefore, advancing the understanding of the dynamics underlying the system justification motivation can shed light on why individuals support ideologies that maintain inequality, providing important implications for theory and practice.

The present research focuses on a topic that has received relatively little attention to date: the relationship between psychological distance and system justification. As defined, psychological distance is a contextual variable with which mental representations of objects, events, actions, goals and other people are associated based on different levels of abstraction (or construal; Trope & Liberman, 2010). The present research aims to determine whether and how, as psychological distance conditions vary, differences in system justification motivation emerge among individuals from groups with different statuses, ideologies and values.

### System justification

According to the System Justification Theory (SJT; Jost & Banaji, 1994), people are motivated to defend, justify and strengthen the existing social, economic and political systems, institutions and arrangements. This suggests that people perceive existing conditions of inequality not only as legitimate but also as natural and necessary. The activation of system justification is influenced by the interaction with two other fundamental motivations: ego justification (i.e. the motivational tendency to defend self‐interests, including a positive self‐image) and group justification (i.e. the motivational tendency to defend the interests of the ingroup, including the positive image of its members). Most frequently, ego, group and system justification motivations coincide and reinforce one another for members of privileged groups: to the extent that privileged individuals defend the system, they also defend the ingroup's privileges and, by extension, personal interests. However, for members of disadvantaged groups, these three motivations frequently conflict with each other: defending the system contradicts both personal and ingroup interests. Thus, system justification is positively related to group and ego justification among members of the dominant group, whereas it is negatively related among members of the subordinate group (Jost & Banaji, 1994).

Furthermore, numerous studies have found that political orientation is associated with differences in system justification, with conservatives generally showing higher levels of system‐justifying tendencies compared to liberals (Jost et al., 2002). Conservative ideology constitutes a prototypical example of a system‐justifying ideology, as it effectively satisfies individuals' epistemic, existential and relational needs (Jost et al., 2003, 2007). Specifically, conservative individuals, compared to liberals, are more inclined to defend the existing social order even in the face of social and economic inequalities, as they tend to perceive such inequalities as legitimate and functional for maintaining the status quo (Jost et al., 2003; Napier & Jost, 2008). This orientation results in a stronger alignment between ego, group and system justification motivations, since defending the societal status quo also entails defending personal and political ingroup interests. In contrast, liberal ideology—more strongly oriented towards social change and the reduction of inequality (Graham et al., 2009)—may lead to a greater conflict between the desire to defend one's ideological ingroup, which often promotes progressive and redistributive goals, and the more general tendency to justify the status quo. In this sense, for liberal individuals, the motivation to justify the system may conflict with identification with an ideological group that challenges structural inequalities and advocates for social change. This conflict highlights the relevance of political orientation in the dynamics of system justification, particularly in relation to the distinct motivations involved (Jost, 2019; Jost et al., 2003).

Alongside this perspective, alternative models have been developed that seek to explain system justification within the frameworks of social identity theory (SIT; Tajfel & Turner, 1979) and self‐categorization theory (SCT; Turner et al., 1987; Turner & Reynolds, 2012). In particular, the Social Identity Model of System Attitudes (SIMSA; Owuamalam et al., 2016; Rubin et al., 2023) proposes that tendencies to legitimize the status quo do not stem from a distinct system justification motive, but can instead be understood as expressions of social identification processes. According to this model, system‐justifying attitudes reflect the salience of different levels of identity: in some circumstances, supporting the system may serve to defend one's ingroup or a broader collective identity, rather than representing an independent motivation. From this perspective, SIMSA highlights that system justification processes depend on which level of identity is made salient, underscoring the importance of considering the different levels of abstraction at which social identity can be represented.

Overall, given the spread of system justification and its role in inequality maintenance, investigating variables that may increase or decrease system justification is of the utmost importance. This is where the current research comes in, as it aims to examine the influence (if any) of psychological distance.

### Construal Level Theory of psychological distance

According to the Construal Level Theory (CLT; Trope & Liberman, 2010), individuals can mentally represent stimuli at various levels of abstraction (or construal), and this process is closely related to psychological distance. Information about a stimulus is processed differently depending on the level of psychological distance, which is the subjective experience of what is distant from the self in the here and now (Liberman & Trope, 2014). Psychologically distant stimuli activate high‐level construals, which are abstract and global, whereas close stimuli elicit low‐level construals, which are concrete and specific (Förster et al., 2004; Freitas et al., 2004). Psychological distance consists of four dimensions: temporal, spatial, social and hypothetical. These dimensions are interrelated and influence one another to produce a unified perception of distance (Maglio et al., 2013a, 2013b). Importantly, the relationship between psychological distance and construal level is bidirectional, meaning that not only does distance influence the level of abstraction, but adopting a certain level of construal can also expand or reduce perceived psychological distance (Bar‐Anan et al., 2006, 2007).

Building on this perspective, it is important to consider how construal level relates to the salience of different forms of self‐definition. Self‐Categorization Theory (Turner et al., 1987; Turner & Reynolds, 2012) posits that individuals can categorize themselves at different levels of abstraction: as unique persons, as members of a group in contrast to others or as part of more inclusive and superordinate identities. The salience of each level is not fixed, but depends on contextual cues and the accessibility of available social categories (Oakes, 1987). Construal Level Theory provides insights for understanding these processes: abstract construals, typically activated by greater psychological distance, are associated with a broader and more inclusive focus, whereas concrete construals, linked to psychological proximity, emphasize individuating features or intergroup boundaries (Liberman & Trope, 2008; McCrea et al., 2012; Wakslak et al., 2006). Overall, the literature suggests that psychological distance can modulate the level of identity that becomes salient, thereby influencing the relative weight of lower‐ versus higher‐level identity considerations. Consequently, system justification tendencies may also vary for individuals belonging to different groups, as a function of the identity level made salient by construal level. These considerations therefore provide the theoretical basis for analysing more specifically the relationship between psychological distance and system justification.

### Psychological distance and system justification

There are currently few studies investigating the relationship between psychological distance and system justification, and findings are mixed: some studies have shown greater legitimization of inequality under conditions of high psychological distance or perceived system stability (Chan, 2016; Laurin et al., 2013; Ledgerwood et al., 2010), suggesting that when individuals perceive the system as distant, abstract or unchangeable, they may be more likely to rationalize existing inequalities as necessary or acceptable. Other studies have found that psychological distance can either reduce or amplify system‐justifying tendencies depending on individuals' preexisting ideological orientations (Alper, 2018; Johnson & Fujita, 2012; Luguri et al., 2012), showing that high‐level construals may strengthen ideological commitment, enhancing system support among conservatives while decreasing it among liberals. Additionally, temporal proximity to system threats has been found to intensify system‐justifying responses among individuals with strong traditionalist beliefs (Miller & Borgida, 2019). Importantly, Luguri and Napier (2013) showed that the effect of construal level depends on which identity is salient: when a partisan identity was made salient, abstract thinking increased polarization between liberals and conservatives, whereas when a national identity was salient, abstract thinking reduced polarization. This work illustrates that abstraction can sometimes reinforce system‐defensive motives and at other times make superordinate concerns more salient. In line with this view, our research situates psychological distance within self‐categorization processes, suggesting that distance may alter the relative salience of different identity levels, which may help explain why psychological distance sometimes reinforces system justification and at other times attenuates it.

Drawing upon SCT, we propose that psychological distance, through the level of cognitive abstraction it activates, may modulate the salience of different levels of identity. Under conditions of low psychological distance, lower‐level identity perspectives are more accessible, whereas greater psychological distance favours the activation of higher‐level, superordinate identities, directing attention towards broader and more inclusive collective considerations. This perspective provides a framework for examining how system justification may vary as a function of identity salience. Our research focuses on differences between groups with opposing status or ideological orientations. Specifically, we aim to investigate whether psychological distance, by shaping the salience of different levels of social identity, leads individuals to prioritize collective interests associated with superordinate identities over those tied to lower‐level identity concerns. Thus, our goal is to examine the conditions under which system justification increases or decreases depending on individuals' group membership—particularly in terms of status and ideology—as a function of the level of social identity made salient by psychological distance.

Important for the current research, previous studies have demonstrated that a high level of abstraction—typically associated with greater psychological distance—leads individuals to focus more on values and goals related to the broader collective, promoting cooperation and integrative decision‐making (Giacomantonio et al., 2010; Henderson, 2011; Henderson et al., 2006; Henderson & Trope, 2009; Stillman et al., 2018). Conversely, a concrete mindset, associated with psychological proximity, tends to emphasize self‐relevant, short‐term concerns and self‐interest (Van Lange & Huckelba, 2021). Thus, when psychological distance is high, higher‐level identities tend to be more prominent, whereas individuals focus more on lower‐level identities when psychological distance is low. The present research aims to extend the findings from studies that contrasted individual interests with those of the group or broader collective. We aim to shift this distinction from the individual level to the intergroup level, focusing on the potential conflict between lower‐level (subgroup) and higher‐level (superordinate) identities. Within this framework, we assume that the collective is more relevant at a higher level of abstraction and that psychological distance can thus modulate the relative salience of motivations directed towards ingroup protection versus those oriented towards the welfare of the broader collective, in line with the idea that different levels of social identity may become salient at different levels of abstraction.

In other words, we propose that, under conditions of low psychological distance, individuals are more likely to focus on lower‐level identity motivations, whereas greater distance favours a more abstract and inclusive representation aligned with higher‐level, superordinate identities (e.g. society as a whole). This shift in identity level may, in some cases, lead to a reduction in system justification, particularly among groups that tend to adopt an ingroup perspective. In this sense, our hypothesis is that psychological distance modulates which level of social identity becomes salient, and thereby the motivations underlying system‐related evaluations. Previous work indicates that abstraction can sometimes strengthen, rather than weaken, hierarchy‐consistent attitudes (Laurin et al., 2013; Ledgerwood et al., 2010). Our perspective therefore focuses on the shift in the level of identity that becomes salient with greater psychological distance, while recognizing that the outcomes of this shift may vary across contexts. Thus, in this paper, we use the term ‘collective concerns’ specifically to denote motivations tied to superordinate identities (e.g. society and nation). Importantly, this does not necessarily imply prosocial or egalitarian motives, but may reflect an identity‐level shift towards superordinate considerations.

Based on these assumptions, we hypothesize that when adopting a low‐level construal (or when psychological distance is low), lower‐level (subgroup) identity motivations prevail. Conversely, when adopting a high‐level construal (or when psychological distance is high), higher‐level, superordinate identity considerations become more prominent. This would indicate that low psychological distance demotivates or motivates individuals to justify the system (and maintain inequality) depending on their status (advantaged vs. disadvantaged) and ideology (conservative vs. liberal). In advantaged groups and among conservatives, group and system justification motives tend to align, fostering a stronger preference for legitimizing the social order, whereas in disadvantaged groups and among liberals, these motives are more likely to conflict. Thus, because lower‐level identity motivations are salient at low psychological distance, disadvantaged group members and liberals would endorse lower system justification. In contrast, advantaged group members and conservatives would endorse higher system justification, because these motivations align with their motivation to maintain the status quo. Conversely, a high psychological distance would motivate individuals to focus on higher‐level, superordinate identities, considering interests that transcend those of one's immediate group. As a result, differences in system justification across groups would likely diminish, with advantaged group members and conservatives, in particular, showing a reduced tendency to legitimize social inequalities. Support for these predictions would highlight the role of psychological distance in shifting the salience of lower‐ (subgroup) versus higher‐level (superordinate) identities, thereby influencing individuals' propensity to justify or challenge social inequalities.

### Research overview

We conducted three experimental studies in which psychological distance was manipulated. Specifically, we chose to manipulate temporal distance, based on three main considerations. First, manipulation of the temporal dimension of psychological distance has been widely employed in the CLT literature as a reliable means of activating different levels of abstraction (Trope & Liberman, 2003, 2010). Second, compared to other forms of psychological distance such as social distance, temporal distance does not directly imply a distinction between groups, making it more suitable for testing the effect of abstraction in a way that is more neutral with respect to intergroup content (see Luguri & Napier, 2013). Third, temporal distance offers the advantage of being manipulated independently of the outcome measures: it is sufficient to activate a near versus distant temporal construal, without altering the content of the issues under evaluation. This makes temporal distance more practical than other dimensions (e.g. hypotheticality or spatial distance), which are harder to operationalize without entangling the manipulation with the outcome variables.

Accordingly, Study 1 investigated whether and how temporal distance affects system justification among disadvantaged and advantaged groups. These groups were defined according to the context of gender inequality (women vs. men). In Study 2, we focused on political orientation and investigated whether and how temporal distance affects system justification among left‐wing vs. right‐wing supporters. Finally, in Study 3, we directly tested whether low temporal distance increases the salience of lower‐level identity concerns, whereas high temporal distance enhances the salience of higher‐level, superordinate, collective identity concerns. To do so, we explored how exposure to threats framed at a lower versus higher level of identity abstraction influenced perceived relevance, emotional distress and coping responses.

Design, method and results for each of the studies are described below. Demographic information and descriptive statistics are reported in detail in the Appendix S1 for each of the studies.

All the studies were conducted in accordance with the Helsinki's Declaration. All participants gave their consent before their inclusion in the study.

All the data and codebooks for this research are available at the following link: https://osf.io/6ajxq/?view_only=7261e90ac4a84987bc38f0f5bd4a0f13.

## STUDY 1

Study 1 investigated the influence of psychological distance in the context of gender inequality. Given that previous research indicates that women are disadvantaged relative to men, particularly in terms of employment, wages and career opportunities (Barreto et al., 2009; Bowles & McGinn, 2004; Eagly & Carli, 2007; Hoyt, 2010), we investigated whether and how psychological distance moderates the relationship between gender and system justification. Specifically, we examined people's motivation to legitimize the gender gap phenomenon, which has been the subject of prior research on system justification (De Cristofaro et al., 2021). In Study 1, we conceptualized the ‘collective’ as a higher‐level, superordinate societal identity (society at large), whose interests may diverge from those of the advantaged group (men). We assumed that low psychological distance would activate lower‐level (subgroup) identity perspectives, leading men to defend the existing gender hierarchy to protect their group's relative advantages. In contrast, at greater distance, abstraction was expected to increase the salience of the broader societal identity, highlighting costs of inequality. Accordingly, we predicted that in the condition of low psychological distance, men would justify the gender gap more than women. In contrast, in the condition of high psychological distance, gender differences in system justification were expected to disappear, as men's tendency to legitimize inequality would be attenuated when higher‐level identities became more salient.

### Method

#### Participants

Data were collected online through the Prolific platform and participants were compensated £1.10 for completing a 15‐min questionnaire. G*Power (Faul et al., 2007) was used to determine the required sample size to perform a two‐way analysis of variance (ANOVA) between groups. Analysis revealed that about 128 participants are required, assuming a statistical power of 0.80, an expected *f* of 0.25 and an alpha probability level of .05. The original sample consisted of 215 Italian participants. Among them, 43.7% identified as men, 53.7% as women, 2.2% as non‐binary and one participant (0.4%) decided not to disclose their gender. Based on our hypotheses, we decided to include only participants who strongly identified as either men or women. Thus, six participants who did not specify their gender as either men or women were excluded from the analyses. The final sample consisted of 209 participants, with a mean age of 29.07 years (SD = 10.22) and an age range of 18–75 years.

#### Procedure

After providing demographic information, participants were randomly assigned to one of two experimental conditions: low psychological distance or high psychological distance. To manipulate psychological distance, participants were asked to imagine what they would do at a moment near in time (i.e. Monday of the following week) or far in time (i.e. a Monday next year) for the low vs. high psychological distance condition, respectively. Participants were invited to describe their activities, emotions and events. This manipulation has been used in previous studies and shown to be effective at inducing low vs. high psychological distance (De Dreu et al., 2009; Giacomantonio et al., 2010). After the manipulation was completed, participants were presented with the manipulation check. Finally, participants were instructed to carefully read a text describing the gender gap phenomenon in Italy, after which their propensity to legitimate the gender pay gap was measured.

#### Measures

##### Manipulation check

To test the validity of the manipulation, participants were asked to reflect on their responses to the task and indicate, for seven semantic differentials, whether they pertained to low or high levels of construal: Insignificant–Significant, Unimportant–Important, Low priority–High priority, Particular–Global, Concerning ‘how’–Concerning ‘why’, Short‐term goals–Long‐term goals and Secondary in life–Central in life (Giacomantonio et al., 2010). These items were averaged and constituted a psychological distance index (*α* = .67, M = 4.53, SD = 0.95) with low scores indicating low distance and high scores indicating high distance.

##### Gender pay gap justification

We used three items (Jost & Burgess, 2000) that asked participants to rate on a scale from 1 (not at all) to 7 (very much) how ‘fair’, ‘justifiable’ and ‘legitimate’ they believed the gender gap to be to determine the extent to which they justified gender inequality in employment and wages. A global gender gap justification index was derived from the mean of the three items (*α* = .82, M = 1.67, SD = 1.14).

### Results

#### Manipulation check

ANOVA revealed that participants assigned to the high distance condition (M = 4.73, SD = 1.01) reported a significantly higher mean at the manipulation check index (higher scores indicate high‐level characteristics) than participants assigned to the low‐distance condition (M = 4.35, SD = 0.87), *F*_(1,207)_ = 8.48, *p* = .004, *η*_*p*_^2^ = 0.04, confirming the effectiveness of the manipulation.

#### Analysis of variance

**FIGURE 1:**
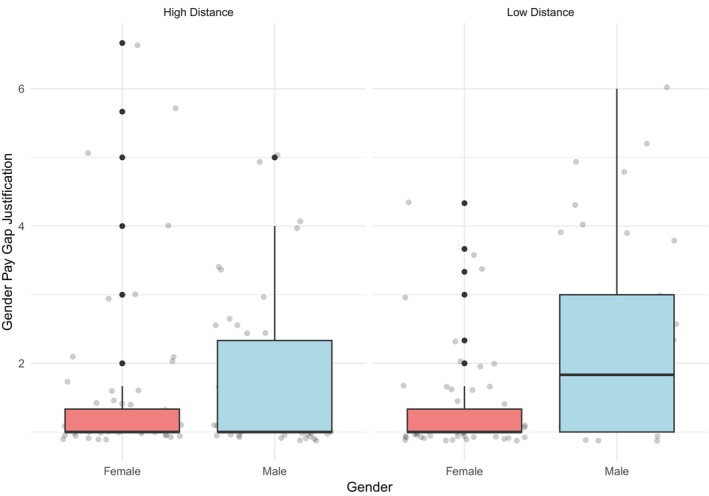
Interaction between gender and psychological distance in gender pay gap justification.

The hypothesized interaction between gender and psychological distance was investigated by conducting a 2 × 2 ANOVA. In line with our hypotheses, results showed a significant interaction between gender and psychological distance (*F*_(3,205)_ = 5.91, *p* = .016, *η* ^2^_*p*_ = 0.03). Pairwise comparisons (Figure 1) revealed a significant difference between men and women showing that men (M = 2.31, SE = 0.17) justify the gender gap more than women (M = 1.30, SE = 0.13) only at low psychological distance (M_Diff_ = 1.01, SE_Diff_ = 0.22, *t* = 4.67, *p* < .001, 95% CI [0.58, 1.43]). In the high psychological distance condition, the difference between men (M = 1.78, SE = 0.16) and women (M = 1.52, SE = 0.15) was not significant (M_Diff_ = 0.26, SE_Diff_ = 0.22, *t* = 1.20, *p* = .231, 95% CI [−0.17, 0.69]). Furthermore, pairwise comparisons indicated that a significant difference in levels of gender gap justification between low and high distance conditions was observed exclusively among men (M_Diff_ = 0.53, SE_Diff_ = 0.23, *t* = 2.28, *p <* .05, 95% CI [0.07, 0.99]). In contrast, this difference was not statistically significant among women (M_Diff_ = −0.21, SE_Diff_ = 0.20, *t* = −1.08, *p* = .28, 95% CI [−0.61, 0.18]).

The main effect of gender was significant (*F*_(3,205)_ = 17.14, *p* < .001, *η* ^2^_*p*_ = 0.08), showing that women (M = 1.41, SD = 0.10) tend to justify the gender gap less than men (M = 2.05, SD = 0.12). Instead, the main effect of the manipulation was not significant (*F*_(3,205)_ = 1.05, *p* = .307, *η* ^2^_*p*_ = .005).

### Discussion

Results of Study 1 supported our hypotheses, demonstrating that psychological distance moderates the relationship between gender and a particular area of system justification: gender pay gap justification. These results show that at low psychological distance, there is a significant gender difference in the level of system justification, with men reporting higher justification of the gender pay gap than women. However, at a high psychological distance, no significant gender difference emerges. As expected, these results suggest that psychological distance may influence the salience of different levels of social identity. Specifically, when psychological distance is low, individuals tend to focus more on lower‐level identity considerations. In this condition, men justify the gender pay gap to a greater extent than women, whose group interests are less aligned with the defence of systemic gender inequalities. In contrast, when psychological distance is high, individuals are more likely to adopt a high‐level construal that emphasizes a superordinate identity and broader collective perspectives, thereby reducing the salience of group‐based interests and, consequently, diminishing gender differences in gender pay gap justification. This appears to be driven by a significant reduction in men's gender pay gap justification levels under high distance conditions, which brings their responses closer to those of women.

## STUDY 2

Building on the findings of Study 1, which focused on gender as a status‐based distinction, Study 2 examined whether similar processes apply when the relevant distinction concerns ideological orientation. Thus, in this study, we compared members of groups with opposing ideologies regarding system justification. The focus was not on the distinction between disadvantaged and privileged groups, but on the distinction between groups that support a non‐legitimating versus legitimating ideology of social inequality. Research suggests that conservatism (or right‐wing political orientation) is a prototypical system‐justifying ideology (Jost et al., 2002). Political orientation may reflect a different relationship between the preference for one's ideological ingroup and the motivation to justify the system. For right‐wing individuals, whose ideology tends to legitimize the existing social order, the interests of the ideological ingroup and those of the system tend to be aligned. In contrast, for left‐wing individuals, who promote a vision centred on social change and the reduction of inequality, the interests of their ideological ingroup may conflict with the motivation to justify the system. Therefore, we investigated the interactive effect of political orientation and psychological distance on system justification. In this context, we conceptualized the ‘collective’ as a higher‐level, superordinate societal identity that transcends partisan divisions. Whereas a low psychological distance was expected to highlight lower‐level ideological identities, a greater distance was expected to make this higher‐level identity salient, thereby reducing the weight of partisan divides. In line with Study 1, we predicted that in the condition of low psychological distance, right‐wing supporters would justify the system more than left‐wing supporters. However, at high psychological distance, right‐wing individuals were expected to reduce their system justification, leading to no significant differences between ideological groups.

### Method

#### Participants

An a priori power analysis was conducted using G*Power to determine the required sample size for detecting a small‐to‐medium interaction effect (*f* ^*2*^ = 0.05) in a linear regression model with three predictors (two main effects and one interaction). Assuming *α* = .05 and power = 0.80, the analysis indicated that a minimum of 159 participants would be required. We collected data from a sample of 350 Italian participants with a mean age of 28.17 years (SD = 8.34), and an age range of 18–62 years. One hundred and eighty‐one participants (51.7%) identified as men, 161 as women (46%), 7 as non‐binary (2%) and 1 was unwilling to answer (0.3%).

#### Procedure

Participants responded to demographic variables, including political orientation. They were then randomly assigned to one of two experimental conditions: low psychological distance or high psychological distance, as in Study 1. Finally, the manipulation check and the tendency to justify the system were measured.

#### Measures

##### Political orientation

A single item asked to indicate the political orientation of the participants on a scale from 1 (Extreme Right) to 7 (Extreme Left) (M = 4.93, SD = 1.20).

##### Manipulation check

Participants responded to the seven semantic differentials used in Study 1 for the manipulation check (*α* = .71, M = 4.22, SD = 0.99).

##### System justification

The General System Justification Scale (Kay & Jost, 2003) was administered to measure participants' tendency to legitimate and defend the system. This scale consists of eight items with response scales ranging from 1 (completely disagree) to 7 (completely agree). Examples include: ‘In general, you find society to be fair’, ‘Most policies serve the greater good’ and ‘Everyone has a fair shot at wealth and happiness’. The index of general system justification was calculated by averaging the items (*α* = .70, M = 4.12, SD = 0.65).

### Results

#### Manipulation check

The ANOVA results demonstrated the effectiveness of the manipulation. Participants in the high‐distance condition (M = 4.41, SD = 0.74) reported a significantly higher mean of the psychological distance index than participants in the low‐distance condition (M = 4.02, SD = 0.74), *F*_(1,348)_ = 14.26, *p* < .001, *η*_*p*_^2^ = 0.04.

#### Moderation analysis

**FIGURE 2:**
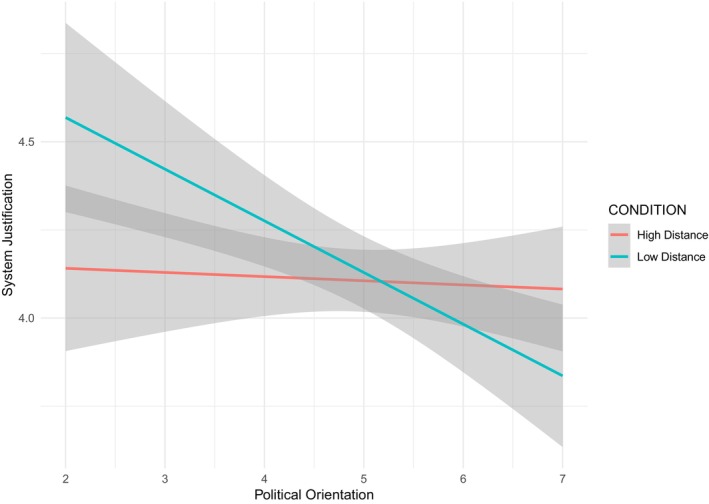
Interaction between political orientation and psychological distance manipulation.

Hypotheses were tested by performing a moderation analysis with SPSS (PROCESS; Model 1, Hayes, 2013) with political orientation as focal predictor, manipulated psychological distance as moderator, and level of system justification as outcome. The model accounted for 4% of the variance in the system justification (*F*_(3,346)_ = 4.61, *p* = .003). As predicted, the interaction was significant (*B* = 0.14, SE = 0.57, *t* = 2.36, *p* = .018, 95% CI [0.02, 0.25]). A simple slopes analysis (Figure 2) revealed that in the low‐distance condition, left‐wing participants justified the system significantly less than right‐wing participants (*B* = −0.15, SE = 0.04, *t* = −3.68, *p* < .001, 95% CI [−0.22, −0.07]), whereas in the high distance condition there was no significant difference (*B* = −0.01, SE = 0.04, *t* = −0.29, *p* = .774, 95% CI [−0.9, 0.68]). Further examination of the interaction revealed that, among right‐wing participants, the difference in system justification levels between low and high psychological distance reached a marginal level of significance (*B* = −0.19, SE = 0.10, *t* = −2.01, *p* = .05, 95% CI [−0.38, −0.01]), suggesting a tendency to justify the system less under conditions of high psychological distance. In contrast, no significant difference in system justification emerged among left‐wing participants as a function of psychological distance (*B* = 0.13, SE = 0.10, *t* = 1.34, *p* = .18, 95% CI [−0.06, 0.32]).

Political orientation was significantly and negatively related to system justification (*B* = −0.28, SE = 0.09, *t* = −3.14, *p* = .002, 95% CI [−0.46, −0.10]), indicating that right‐wing participants tend to justify the system more than left‐wing participants, consistent with the existing literature (Jost et al., 2003). The main effect of psychological distance on system justification was not significant (*B* = −0.03, SE = 0.07, *t* = −0.47, *p* = .637, 95% CI [−0.17, 0.10]).

### Discussion

Consistent with Study 1, these results showed that in the condition of low psychological distance, right‐wing individuals justified the system more than left‐wing individuals, supporting our hypothesis. This is also consistent with the notion that when psychological distance is low, individuals tend to rely more on lower‐level identity considerations, in line with a focus on subgroup‐based rather than superordinate identities. We also found that there were no significant differences in system justification between left‐ and right‐wing individuals in the condition of high psychological distance. This convergence appeared to be driven by a relative reduction in system justification among right‐wing participants under high psychological distance, whose levels became more similar to those of left‐wing participants, possibly reflecting a shift from lower‐level to higher‐level identity considerations.

These findings also contribute to the ongoing debate on the role of construal level in political polarization. Prior studies have shown that high‐level construals can either intensify or attenuate polarization depending on which identity is made salient (Ledgerwood et al., 2010; Luguri et al., 2012; Napier & Luguri, 2016; Yang et al., 2013). For example, Luguri and Napier (2013) showed that when participants' political identity was activated, greater abstraction increased polarization between liberals and conservatives, whereas when national identity was activated, greater abstraction reduced polarization. In contrast to these studies, our research relied on a neutral manipulation of psychological distance that was not tied to a specific social or political identity. Under these conditions, our results indicate that abstraction generally reduced polarization in system justification, as right‐wing and left‐wing individuals converged under high psychological distance. Thus, our findings suggest that when no specific identity is made salient, psychological distance may help to diminish partisan divides by increasing the salience of superordinate collective perspectives, thereby attenuating ideological polarization in system justification.

However, it should be noted that political orientation was measured with a single item self‐placement on a left–right continuum. Although this measure is widely used in political psychology and remains a common indicator of political orientation in European contexts, including Italy (Jost, 2006; Jost et al., 2009), it may not fully capture the multidimensional nature of ideological orientations. Future research would benefit from employing more comprehensive measures of political ideology to assess whether our findings generalize across different dimensions of conservatism and liberalism (e.g. economic vs. cultural).

It is also important to note that the interaction observed in this study was relatively small and the pattern of conditional effects was not uniformly robust. This is not unexpected, given that system justification is a complex construct shaped by multiple social and individual factors beyond political orientation (Jost, 2019). Nevertheless, the finding that psychological distance moderated ideological differences, even modestly, provides evidence for the role of abstraction in affecting different system‐legitimizing attitudes.

Overall, Studies 1–2 show that under conditions of low psychological distance, individuals tend to rely more on lower‐level identities. In contrast, under conditions of high psychological distance, individuals are more inclined to place less emphasis on the ingroup's interest and shift their focus to higher‐level, superordinate collective concerns and the broader societal implications of inequality. To more directly capture this shift in identity salience, Study 3 moved beyond group‐based comparisons and examined responses to threats framed at lower versus higher levels of identity abstraction. By comparing reactions to lower‐level versus higher‐level threats under different conditions of psychological distance, Study 3 provides a clearer test of whether psychological distance alters the predominance of less inclusive versus more inclusive identity‐based considerations.

## STUDY 3

While Studies 1 and 2 indicated that psychological distance moderates group‐ and ideology‐based differences in system justification, Study 3 was designed to explore the underlying mechanism by testing whether psychological distance shifts the salience between identities with different levels of abstraction. Specifically, it examined whether lower‐level versus higher‐level identity concerns become more influential under low versus high psychological distance. We directly tested the hypothesis that under conditions of low psychological distance, lower‐level identity concerns become more salient, whereas under conditions of high psychological distance, individuals are more motivated by higher‐level, superordinate identity considerations related to the broader social system. This study adopted a different approach compared to the previous ones, in which higher psychological distance reduced the salience of subgroup‐based differences. In contrast, here we designed the study so that differences between conditions would become more pronounced at high psychological distance. To achieve this, we shifted the focus from group‐level comparisons to threats framed at lower versus higher levels of identity abstraction, allowing us to directly compare the influence of lower‐level versus superordinate‐level identity concerns. To compare these circumstances, we utilized scenarios that framed the threat at a lower versus a higher level of identity abstraction and examined whether, under different conditions of psychological distance, the impact of one threat is greater than the other. In this study, we conceptualized the ‘collective’ as the superordinate national identity, which represents a level of self‐categorization beyond the subgroup (Gaertner et al., 2016; Turner et al., 1987). Thus, whereas the lower‐level threat referred to concerns framed at the most concrete level of identity (personal), the collective threat was designed to invoke concerns tied to the national community as a whole. It is important to note that, unlike the previous studies, Study 3 did not include a direct measure of system justification. This was a deliberate methodological choice, as the aim was not to assess system‐legitimizing attitudes per se, but to provide a more direct test of the proposed mechanism by examining whether psychological distance regulates the salience of different levels of identity. Accordingly, we focused on participants' subjective responses to the threat scenarios, including their perceived relevance, emotional reactions and coping intentions, as indicators of whether psychological distance shifts attention across different levels of identity.

Based on previous research and on the results from Studies 1–2, we predicted that under conditions of low psychological distance, the threat framed at a lower level of identity abstraction would be more influential than the threat framed at a higher, collective level in terms of relevance, negative emotional reactions and coping intentions. Conversely, under conditions of high psychological distance, the collective (higher‐level) threat would be more influential than the lower‐level threat.

### Method

#### Participants

The required sample size for this study was calculated using the same a priori power analysis procedure as in Study 1. The sample consisted of 244 participants, of which 52% identified as men, 42% as women, 3.3% as non‐binary and 0.8% did not wish to specify their gender. The sample ranged in age from 19 to 65 years (M = 29.18, SD = 8.80).

#### Procedure

Participants initially provided demographic information and were randomly assigned to either the low or high psychological distance conditions, as in Studies 1 and 2. After completing the manipulation check items, participants were randomly assigned to read a threat scenario framed at either a lower or a higher level of identity abstraction. Participants in the lower‐level threat condition read a passage about the health risks of cell phone use (Van Der Toorn et al., 2017). The text referred to scientific evidence linking cell phone exposure to problems with sleep, mood, learning, fertility, hearing, vision and even increased risks of tumours and neurodegenerative diseases. Participants in the collective threat condition read a passage highlighting Italian citizens' dissatisfaction with the country's condition (Kay et al., 2005). In particular, the text emphasized widespread political instability, economic decline and a general sense of insecurity, including references to citizens' intentions to emigrate. These scenarios were selected because they represent prototypical threats situated at different levels of identity abstraction. The cell phone scenario highlights risks framed at a lower, more concrete level of identity, whereas the national crisis scenario involves threats to the country as a whole, reflecting concerns at a higher, collective, superordinate level. Although the two scenarios differ in content, both appropriately express the contrast between threats framed at lower versus higher levels of identity abstraction that was central to our research question. After reading the threat scenario, participants completed manipulation check items. Finally, they rated the significance of the threat they had been exposed to, the emotional distress it induced, and their propensity to take action to find a solution.

#### Measures

##### Manipulation check

After the psychological distance manipulation, participants rated their task responses on the seven semantic differentials used in our previous studies (*α* = .66, M = 4.11, SD = 0.92).

##### Threat manipulation check

To assess the efficacy of the threat manipulation, participants rated 12 items on response scales ranging from 1 (not at all) to 7 (completely) to indicate the extent to which they perceived threats framed at different levels of identity abstraction. Specifically, 6 items were administered to measure perceptions of threat at a lower, more concrete level of identity: ‘I feel threatened’, ‘My personal condition is at risk’, ‘I personally feel I am in danger’, ‘I feel that my personal condition is in danger’, ‘I perceive a sense of personal threat’ and ‘If I think about myself, I do not feel safe’. The 6 higher‐level threat items were: ‘The Italian system is threatened’, ‘The condition of my country is at risk’, ‘In general, society is in danger’, ‘The condition of my country is in danger’, ‘I perceive a sense of threat to society’ and ‘If I think about the Italian system, I feel that society is not safe’. The index of lower‐level threat (*α* = .94, M = 3.25, SD = 1.34) and higher‐level threat (*α* = .95, M = 3.95, SD = 1.50) was calculated by averaging the items.

##### Threat relevance

On a seven‐point scale ranging from 1 (not at all) to 7 (completely), participants rated the passage they had just read as relevant, salient, important, significant, interesting, marginal (reverse‐scored) and negligible (reverse‐scored). The items were averaged to determine the threat relevance index (*α* = .94, M = 5.24, SD = 1.26).

##### Negative emotional impact

Participants were asked to recall the passage they had just read and indicate, on a scale ranging from 1 (not at all) to 7 (completely), how strongly they experienced uneasiness, discomfort, anxiety, sadness, agitation, anger, joy (reverse‐scored) and indifference (reverse‐scored). The items were averaged to create a negative emotional impact index (*α* = .89, M = 4.30, SD = 1.25).

##### Coping response

Participants responded to 5 items with response scales ranging from 1 (not at all) to 7 (completely), which asked how much they were willing to engage in behaviours to obtain information and identify solutions to what they had just read. The five items were: ‘I would like to learn more’, ‘I would like to find a solution’, ‘I would attend educational meetings on the topic’, ‘I would take actions that are useful to change’ and ‘I would work to make the situation better’. A coping response index (*α* = .92, M = 4.52, SD = 1.46) was derived by the items' mean.

### Results

#### Manipulation check

Analysis of variance revealed that participants assigned to the high distance condition (M = 4.25, SD = 0.83) reported a significantly higher mean at the check variable (where high scores indicate high‐level characteristics) than those assigned to the low‐distance condition (M = 3.97, SD = 0.83), *F*_(1,242)_ = 5.51, *p* = .020, *η* ^2^_*p*_ = .02.

##### Threat manipulation check

Regarding threat manipulation, a One‐way ANOVA revealed that the perception of the higher‐level threat (M = 3.95, SD = 1.97) was more intense than the perception of the lower‐level threat (M = 3.25, SD = 1.34), *F*_(1,242)_ = 56.38, *p* < .001, *η* ^2^_*p*_ = 0.19. In addition, a significant two‐way interaction emerged between exposure to lower‐level vs. higher‐level threat and self‐reported threat perception, *F*_(1,242)_ = 86.80, *p* < .001, *η* ^2^_*p*_ = 0.26. In the condition of lower‐level threat, the mean perception of threat at a lower identity level was greater (M = 3.42, SD = 1.30) than the mean perception of threat at a higher identity level (M = 3.11, SD = 1.36). In contrast, in the condition of higher‐level threat, scores of the perceived threat at a higher identity level (M = 4.58, SD = 1.32) were greater than the scores of the perceived threat at a lower identity level (M = 3.26, SD = 1.37). As such, the threat manipulations were effective.

##### Dependent variables

A Two‐way ANOVA to test the interaction between psychological distance and type of threat was conducted for each dependent variable. Results are discussed below; means and pairwise comparisons for each dependent variable can be seen in Table 1.

**TABLE 1:** Pairwise comparisons for threat relevance, negative emotional impact and coping response.

Dependent variable	Comparison	M_i_ (SE)	M_j_ (SE)	Diff_i‐j_	SE	*t*	*p*	95% C.I.	
								Lower	Upper
Threat relevance	Thr_low − (dist_low vs. dist_high)	**5.45 (0.16)**	**4.73 (0.16)**	**0.722**	**0.227**	**3.185**	.**002**	**0.276**	**1.17**
	Thr_high − (dist_low vs. dist_high)	5.20 (0.15)	5.60 (0.15)	−0.403	0.216	−1.867	.063	−0.828	0.022
	Dist_low − (Thr_low vs. Thr_high)	5.45 (0.16)	5.20 (0.15)	0.251	0.222	1.132	.259	−0.186	0.687
	Dist_high − (Thr_low vs. Thr_high)	**4.73 (0.16)**	**5.60 (0.15)**	**−0.875**	**0.221**	**−3.954**	**<.001**	**−1.310**	**−0.439**
Negative emotional impact	Thr_low − (dist_low vs. dist_high)	**4.52 (0.15)**	**3.38 (0.15)**	**1.140**	**0.211**	**5.410**	**<.001**	**0.725**	**1.556**
	Thr_high − (dist_low vs. dist_high)	**4.39 (0.14)**	**4.86 (0.14)**	**−0.471**	**0.201**	**−2.346**	.**020**	**−0.866**	**−0.075**
	Dist_low − (Thr_low vs. Thr_high)	4.52 (0.15)	4.39 (0.14)	0.127	0.206	0.619	.537	−0.278	0.533
	Dist_high − (Thr_low vs. Thr_high)	**3.38 (0.15)**	**4.86 (0.14)**	**−1.484**	**0.206**	**−7.215**	**<.001**	**−1.889**	**−1.079**
Coping response	Thr_low − (dist_low vs. dist_high)	**4.85 (0.19)**	**3.98 (0.19)**	**0.863**	**0.265**	**3.255**	.**001**	**0.341**	**1.385**
	Thr_high − (dist_low vs. dist_high)	4.42 (0.18)	4.84 (0.18)	−0.417	0.252	−1.651	.100	−0.914	0.080
	Dist_low − (Thr_low vs. Thr_high)	4.85 (0.19)	4.42 (0.18)	0.424	0.259	1.638	.103	−0.086	0.934
	Dist_high − (Thr_low vs. Thr_high)	**3.98 (0.19)**	**4.84 (0.18)**	**−0.855**	0.**259**	**−3.308**	.**001**	**−1.364**	**−0.346**

Abbreviations: Dist_high, high psychological distance condition; Dist_low, low psychological distance condition; Thr_high, higher‐level threat; Thr_low, lower‐level threat.

##### Threat relevance

**FIGURE 3:**
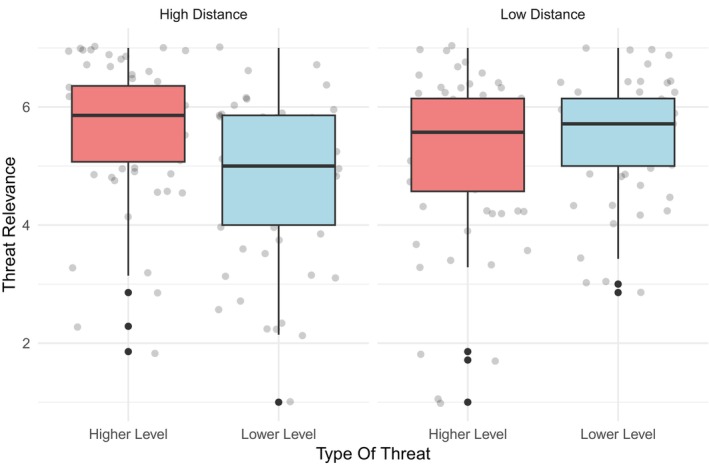
Two‐way interaction between psychological distance and threat on threat relevance.

The interaction between psychological distance and threat was significant (*F*_(3,240)_ = 12.92, *p* < .001, *η* ^2^_*p*_ = 0.05). Pairwise comparisons (Figure 3) revealed that the difference between groups means was significant only in the high distance condition (*p* = .001, see Table 1), where participants exposed to the higher‐level threat attributed greater relevance than participants exposed to the lower‐level threat, and not in the low‐distance condition (*p* = .26). Moreover, participants in the lower‐level threat condition rated the threat as more relevant under low compared to high psychological distance (*p <* .01, see Table 1), whereas no significant difference in perceived relevance emerged for the higher‐level threat as a function of psychological distance (*p* = .06). A main effect of threat emerged (*F*_(3,240)_ = 3.97, *p* = .047, *η* ^2^_*p*_ = 0.02), indicating that participants assigned to the higher‐level threat condition rated the text as more relevant than those in the lower‐level threat condition. The main effect of psychological distance was not significant (*F*_(3,240)_ = 1.04, *p* = .309, *η* ^2^_*p*_ = 0.09).

##### Negative emotional impact

**FIGURE 4:**
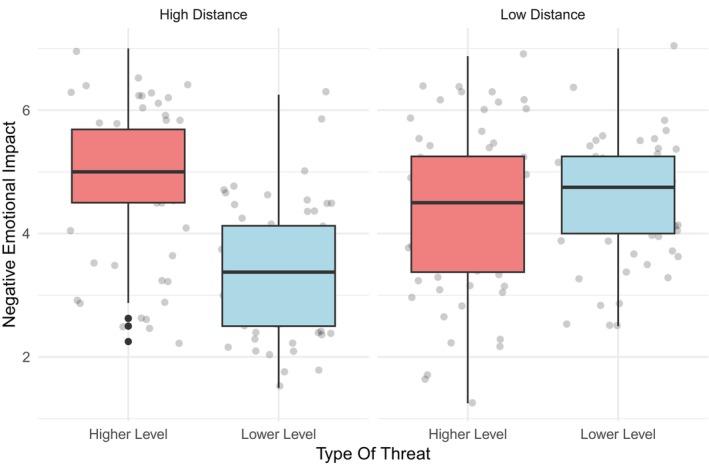
Two‐way interaction between psychological distance and threat on negative emotional impact.

The interaction between psychological distance and threat was significant (*F*_(3,240)_ = 30.64, *p* < .001, *η* ^2^_*p*_ = 0.11) Pairwise comparisons (Figure 4) revealed that the interaction was significant in the high distance condition (*p* < .001) but not in the low‐distance condition (*p* = .537). Lower‐level threat was associated with greater negative emotions under conditions of low psychological distance (*p* < .001), whereas higher‐level threat was associated with greater negative emotions under conditions of high psychological distance (*p* < .05). The conducted analysis revealed that the main effects of both psychological distance (*F*_(3,240)_ = 5.29, *p* < .05, *η* ^2^_*p*_ = 0.02) and threat (*F*_(3,240)_ = 21.72, *p* < .001, *η* ^2^_*p*_ = 0.08) on emotional impact were significant. Specifically, participants assigned to the low‐distance condition exhibited more intense negative emotions than those assigned to the high‐distance condition. Participants exposed to the higher‐level threat condition experienced a greater negative impact than those exposed to the lower‐level threat condition.

##### Coping response

**FIGURE 5:**
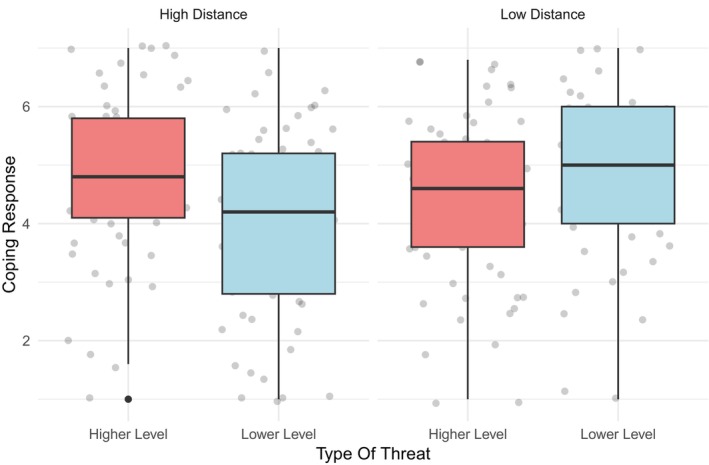
Two‐way interaction between psychological distance and threat on coping response.

The interaction between psychological distance and threat was significant (*F*_(3,240)_ = 12.22, *p* = .001, *η* ^2^_*p*_ = 0.05). Pairwise comparisons (Figure 5) revealed that the interaction was significant in the high‐distance condition (*p* < .01), but not in the low‐distance condition (*p* = .103). For lower‐level threat, greater coping responses were reported by participants in the low psychological distance condition compared to those in the high‐distance condition (*p* = .001), whereas no significant differences emerged for higher‐level threat as a function of psychological distance (*p* = .10). Neither the main effect of psychological distance (*F*_(3,240)_ = 1.49, *p* = .24, *η* ^2^_*p*_ = 0.01) nor the main effect of threat (*F*_(3,240)_ = 1.39, *p = *.22, *η* ^2^_*p*_ = .01) were significant.

### Discussion

Our hypotheses were only partially supported by the results of this study. Although the pattern of means was as expected, only the differences between the means for the high‐distance condition were statistically significant for all three dependent variables. We found that when participants adopted a high psychological distance, the higher‐level threat had a significantly greater impact than the lower‐level threat. Specifically, greater relevance was attributed to the higher‐level threat; negative emotions were more intense, and the intention to respond to the threat was stronger. Moreover, across all three dependent variables, we consistently found that the impact of the lower‐level threat was perceived as stronger under conditions of low compared to high psychological distance. In contrast, only negative emotions associated with the higher‐level threat were reported as more intense under high compared to low psychological distance.

In the low‐distance condition, pairwise comparisons revealed that the lower‐level threat tended to be perceived as more relevant, to cause greater negative emotional impact and to promote coping responses to a greater extent than the higher‐level threat. However, these differences were not statistically significant. This may be because we did not account for the possibility that, for some participants, the higher‐level threat may also be perceived as personally relevant. Consequently, some participants may have viewed both threats as similarly relevant, which would have prevented either threat from emerging clearly. Another possibility is that the two scenarios were not perfectly balanced in terms of content, urgency and emotional tone, as the national crisis scenario may have been inherently more salient or evocative than the health‐risk scenario. This potential asymmetry could have amplified higher‐level reactions beyond the identity dimension per se. Future research could include measures of the perceived imbalance of the scenarios to provide an additional check on the effectiveness of the manipulation.

In addition, research on fear appeals in health promotion campaigns has demonstrated that conveying messages emphasizing the risks of behaviour and, in general, communicating threatening health information may elicit defensive responses such as denial and avoidance, resulting in ignoring the threat's effects (Ruiter et al., 2014; Van't Riet & Ruiter, 2013).

An alternative approach for future research could involve autobiographical recall manipulations, in which participants are asked to describe situations where they felt threatened at a lower identity level versus threatened at a higher, more superordinate identity level. Such methods could offer alternative ways to test the role of psychological distance in shaping identity‐level salience, thereby strengthening the robustness of the findings.

Overall, the results of Study 3 only partially supported our hypotheses. While the pattern of means was generally consistent with the idea that psychological distance shifts attention from lower‐ to higher‐level identity concerns, some effects failed to reach significance. Thus, the evidence should be interpreted with caution. Nevertheless, the finding that the higher‐level threat exerted a stronger influence under high psychological distance suggests a possible mechanism behind the effects observed in Studies 1 and 2. In this sense, Study 3 offers preliminary indications that psychological distance may regulate the salience of different identity levels, though further research is needed to substantiate this interpretation.

## GENERAL DISCUSSION

The overarching purpose of this research was to investigate the relationship between psychological distance and system justification. We conducted three studies in which we experimentally manipulated psychological distance to determine whether and how it moderates the relationship between membership in groups (of opposite (dis)advantaged status and political ideology) and system justification. Building on Self‐Categorization Theory, we reasoned that psychological distance may shift the salience of different identity levels. In line with this assumption, we hypothesized and obtained evidence suggesting that, under conditions of low psychological distance, lower‐level identities become more salient, whereas, under conditions of high psychological distance, higher‐level, superordinate collective identities become more prominent. This shift appeared to be associated with reduced system justification among typically high‐justifying groups and a convergence in attitudes across ideological orientations and different status groups. The operationalization of ‘collective’ was consistent with our broader framework: across studies, the ‘collective’ always refers to a superordinate identity that becomes more salient under greater psychological distance. While the specific referent varied depending on the context, in each case it captured a higher‐order category that transcends immediate subgroup identities, thereby preserving a coherent theoretical meaning.

Specifically, Study 1 revealed that in the context of gender inequality, individuals who are part of the advantaged group (men) were more likely to justify the system than individuals who are part of the disadvantaged group (women) under low‐distance conditions. In contrast, under conditions of high distance, men's justification levels tended to decrease, becoming more similar to those of women. This pattern suggests that greater psychological distance may increase the salience of a more inclusive, superordinate collective identity (e.g. society as a whole), thereby attenuating the typical status‐based differences in system‐legitimizing beliefs.

In Study 2, political ideology‐based distinctions in system justification were examined, revealing that under low‐distance conditions, right‐wing individuals justified the system more than left‐wing individuals; whereas, under high‐distance conditions, right‐wing participants' justification levels tended to decrease, aligning more closely with those of left‐wing participants. These findings point to a reduction in the usual ideological asymmetry, consistent with the idea that greater psychological distance makes superordinate concerns more salient and may attenuate political polarization.

Taken together, these studies suggest that psychological distance may influence individuals' system‐justifying tendencies by moderating the salience of lower‐ versus higher‐level identity considerations, thereby affecting how people perceive and respond to social inequalities across different status and ideological groups. Under conditions of low (vs. high) psychological distance, lower‐level identity concerns appear to be more prominent than higher‐level considerations. In this context, disadvantaged members (Study 1) and left‐wing individuals (Study 2) tended to oppose the legitimization of the status quo, whereas privileged members (Study 1) and right‐wing individuals (Study 2) were more likely to endorse it, as these positions aligned with their subgroup interests. In contrast, under conditions of high psychological distance, system justification seemed less driven by ingroup‐based motives and more consistent with superordinate, collective considerations. As a result, members of privileged groups and right‐wing individuals showed a relative reduction in system‐justifying tendencies, leading to greater convergence with disadvantaged and left‐wing participants. This pattern suggests that psychological distance can shift attention from lower‐level (subgroup) to higher‐level superordinate identities, thereby attenuating ideological and status‐based differences in legitimizing social inequality.

Finally, Study 3 provided partial support for the pattern observed in Studies 1 and 2 by examining how lower‐ versus higher‐level identity threats interact with psychological distance to determine the conditions under which individuals prioritize lower‐level over higher‐level identity concerns—or vice versa. Consistent with Studies 1 and 2, we found that, under high‐distance conditions, participants exposed to a higher‐level, collective threat tended to rate it as more relevant, reported more intense negative emotions and indicated greater willingness to act to find solutions compared to those exposed to a lower‐level threat. Notably, while lower‐level threats were generally perceived as more impactful under low psychological distance, higher‐level threats elicited stronger emotional responses when psychological distance was high, providing evidence that psychological distance may influence the salience and affective weight of different levels of identity.

### Implications

The current research contributes to the advancement of existing literature by providing new evidence regarding the conditions under which system justification can be diminished or heightened. First, this research adds knowledge on the relationship between psychological distance and system justification by suggesting that psychological distance moderates the extent to which disadvantaged and privileged groups justify the system, taking gender‐based groups into account. In general, our findings are consistent with research indicating that system justification tends to increase as advantage increases (Brandt, 2013; Caricati, 2016; Caricati & Lorenzi‐Cioldi, 2012; Owuamalam & Spears, 2020; Trump & White, 2018; Vargas‐Salfate et al., 2018). However, our studies revealed that under conditions of high psychological distance, levels of system justification among privileged group members tended to decrease, resulting in no significant difference compared to disadvantaged individuals. This suggests that system justification may not always increase with social advantage, particularly when lower‐level identity motivations are less salient; rather, status‐based differences in system justification may depend upon contextual factors such as which level of identity is salient. From this perspective, our findings are also consistent with the Social Identity Model of System Attitudes (Owuamalam et al., 2016; Rubin et al., 2023), which complements system justification theory by showing that legitimizing tendencies can often be understood as expressions of social identification processes, depending on which level of identity is activated.

A further implication is that our results were replicated both when status differences and political orientation differences were considered, suggesting a similar psychological mechanism across diverse groups with different characteristics, interests and values. Moreover, in Study 2, we found that the ideological gap between left‐wing and right‐wing individuals was attenuated under high‐distance conditions. These findings are consistent with previous studies that have demonstrated that psychological distance can mitigate differences between liberals and conservatives (Chan, 2016; Luguri et al., 2012; Mahfud et al., 2017; Yang et al., 2012; Yogeeswaran & Dasgupta, 2014). Our findings also speak to the broader literature on group status and political conservatism as motivated resistance to change and to challenges to the status quo (Jost et al., 2003; Knowles et al., 2014; Napier & Jost, 2008). High‐status and conservative groups often benefit from existing arrangements and may therefore be motivated to downplay or ignore information about inequality (see also Apfelbaum et al., 2008; Norton et al., 2006). From this perspective, the tendency of advantaged groups and conservatives to justify the system under low psychological distance can be understood as part of a broader pattern of resisting change that threatens their relative advantages. At the same time, our results suggest that when psychological distance increases and a superordinate, higher‐level identity becomes more salient, these motivations may be tempered, leading advantaged and conservative group members to place relatively more weight on the broader societal costs of inequality.

Although our hypotheses were only partially supported in Study 3, we found that, under high distance conditions, individuals tended to prioritize higher‐level identity considerations over lower‐level ones, in line with evidence linking psychological distance with a greater interest in the larger social and collective unit (Giacomantonio et al., 2010; Henderson, 2011; Henderson et al., 2006; Henderson & Trope, 2009; Stillman et al., 2018).

Beyond its theoretical relevance, our findings also offer potential practical implications. In particular, the evidence that psychological distance can attenuate status‐ and ideology‐based differences in system justification suggests that communication strategies which emphasize more abstract, long‐term or superordinate perspectives might reduce polarization in responses to inequality. For example, framing social issues in terms of their implications for society as a whole, rather than for specific groups, could decrease resistance among advantaged or right‐wing individuals who typically show higher levels of system justification. At the same time, these applications should be approached with caution. As prior research indicates, abstract construals do not invariably promote progressive outcomes (Laurin et al., 2013; Ledgerwood et al., 2010; Luguri & Napier, 2013), and psychological distance may under certain conditions reinforce rather than challenge the legitimacy of existing systems. Thus, our findings should be interpreted as evidence for a conditional effect of psychological distance on system justification, rather than as support for a general tendency whereby abstraction consistently promotes more egalitarian orientations. Accordingly, any practical use of distance‐based framing must carefully consider contextual factors, target audiences and the potential for unintended effects.

### Limitations and future directions

Current research is subject to general limitations. First, the variables were measured using self‐reported instruments, which, although widely employed and validated in the literature, do not allow for controlling for social desirability effects. Exclusive reliance on self‐reports also restricts the ecological validity of our conclusions, as these measures may capture stated beliefs more than actual behaviours. Incorporating behavioural or implicit indicators in future studies would strengthen the robustness of the findings.

A second limitation relates to the generalizability of the results. Given that our samples consist solely of Italian participants, it is necessary to replicate the findings with representative samples from other nations characterized by different socio‐political structures to test the generalizability of the psychological mechanisms observed (e.g. in more collectivist vs. individualist societies) (Price & Murnan, 2004). Indeed, constructs such as political ideology, threat perception and system legitimacy are culturally situated and may carry different implications depending on the historical and institutional context. Cross‐national research has demonstrated that system justification processes are not uniform but vary across cultural orientations and political systems (Jost et al., 2015; Vargas‐Salfate et al., 2018), underscoring the need to test whether the patterns reported here extend beyond the Italian context. Nevertheless, the Italian context, which is marked by political polarization and limited institutional trust, offers an interesting setting for investigating these processes. In this sense, the Italian data should be seen as a useful first step, with future studies needed to determine the extent of their generalizability across different cultural contexts.

The current research is also subject to design‐related limitations, concerning the manipulation of psychological distance. Although manipulating temporal construal is a well‐established approach (De Dreu et al., 2009; Giacomantonio et al., 2010; Trope & Liberman, 2010), its effects may be relatively fragile and subject to decay over the course of demanding experimental tasks. This implies that the manipulation may have provided a conservative test of our hypotheses, such that the effects we observed are likely to represent lower‐bound estimates of the true impact of psychological distance. Another consideration pertains to the size of the effects observed, which were modest. While this is not unusual in research on social and ideological phenomena (Funder & Ozer, 2019; Prentice & Miller, 1992), it suggests that the influence of psychological distance on system justification is likely to be contingent on contextual and individual factors that future research should explore. In this sense, the explanatory power of psychological distance appears limited: its impact on system justification seems to be subtle and context‐dependent rather than that of a strong determinant.

A further limitation is that the present research did not include direct measures of the processes underlying the observed effects. Nevertheless, our results provide a basis for examining more directly these mechanisms. The pattern of findings was coherent across studies and aligned with existing evidence linking psychological distance and abstract construals to shifts in identity salience (e.g. McCrea et al., 2012). Although these mechanisms were not directly measured here, our results are consistent with this perspective and provide a useful starting point. Future research could build on this evidence to examine more explicitly the pathways through which psychological distance influences system‐justifying tendencies. At the same time, future studies could also examine alternative explanations, such as the possibility that reduced system justification under high psychological distance partly reflects diminished emotional engagement or cognitive detachment (Moran & Eyal, 2022; Trope & Liberman, 2010). Future research could more explicitly test these mechanisms by considering direct measures of identity salience and experimental manipulations of identity level (e.g. making subgroup vs. superordinate identities salient) to examine whether these shifts account for the effects of psychological distance. In addition, incorporating indicators of cognitive detachment would help determine whether psychological distance also operates through diminished affective engagement.

Furthermore, our perspective centred primarily on identity‐based processes, in line with the main aims of the present research, without considering other motivational antecedents of system justification. Well‐established factors such as need for cognitive closure, threat sensitivity and uncertainty reduction (De Cristofaro, Giacomantonio, et al., 2022; De Cristofaro, Pellegrini, et al., 2022; Jost et al., 2003, 2007) were not assessed in our studies, leaving open the possibility that psychological distance operates partly by modulating these motives. Future research could investigate whether the effects of psychological distance are mediated by such motives, or whether distance and these motivational factors exert independent or interacting influences on system justification.

Finally, although we found an effect on a particular type of social disadvantage (i.e. gender‐based) associated with a particular type of inequality, additional research is required to determine whether these results are also applicable to other manifestations of inequality, thereby considering other dimensions of social stratification, such as ethnicity and socioeconomic status. Relatedly, numerous studies have demonstrated that system justification can undermine motivation to act collectively against inequality (De Cristofaro et al., 2023; De Cristofaro, Giacomantonio, et al., 2022; De Cristofaro, Pellegrini, et al., 2022; Jost et al., 2017; Osborne et al., 2018; Osborne & Sibley, 2013). Therefore, future research could examine the effects identified in our studies by extending them to collective action for social change (Van Zomeren et al., 2008; see also Scarci et al., 2026).

Overall, future research should further investigate psychological distance as a contextual factor capable of shifting individuals' focus—from lower‐level identity considerations towards higher‐level, superordinate identity concerns—thereby identifying the conditions under which both disadvantaged and privileged individuals become more likely to cope with inequality and no longer legitimize it.

## AUTHOR CONTRIBUTIONS

**Federica Scarci:** Conceptualization; investigation; writing – original draft; methodology; formal analysis; data curation; writing – review and editing. **Matteo Bonora:** Conceptualization; investigation; writing – review and editing; formal analysis; data curation. **Valeria De Cristofaro:** Conceptualization; investigation; writing – review and editing; methodology; data curation. **Valerio Pellegrini:** Conceptualization; investigation; writing – review and editing; formal analysis; data curation. **Mauro Giacomantonio:** Conceptualization; investigation; methodology; writing – review and editing; supervision.

## FUNDING INFORMATION

This research did not receive any specific grant from funding agencies in the public, commercial or not‐for‐profit sectors.

## CONFLICT OF INTEREST STATEMENT

The authors have no relevant financial or non‐financial interests to disclose.

## ETHICS STATEMENT

All methods and procedures were in accordance with the ethical standards of the institutional/national research committee and the 1964 Helsinki Declaration and its later amendments or comparable ethical standards. The study was approved by the Ethical Committee of Sapienza University of Rome [CERT_18FD4F7B951].

## Supporting information


Appendix S1.

## Data Availability

Datasets and Codebooks for this research are available at: https://osf.io/6ajxq/?view_only=cc3bab18289b4b0aacc2f8a2f0466caa.
