# Supplementary material for: From the perspective of the Construal Level Theory: Examining the effect of psychological distance on system justification
Source: Br J Soc Psychol. 2026 Jan 29;65(2):e70047. doi: 10.1111/bjso.70047 (PMC12853408; doi:10.1111/bjso.70047)
Supplement: Supplementary file 1 — Appendix S1. [file BJSO-65-0-s001.docx]

APPENDIX

*Descriptive of the sample’s characteristics (Study 1; N = 209)*

| **Variable** | ***Frequency*** | ***Percentage*** | ***M*** | ***SD*** |
| --- | --- | --- | --- | --- |
| Education |  |  | 2.86 | .94 |
| Middle school diploma | 3 | 1.4% |  |  |
| High school diploma | 87 | 41.6% |  |  |
| Bachelor’s degree | 68 | 32.5% |  |  |
| Master’s degree | 38 | 18.2% |  |  |
| Postgraduate level | 13 | 6.2% |  |  |
| Other | 0 | 0% |  |  |
| Employment |  |  | 1.70 | .74 |
| Student | 98 | 46.9% |  |  |
| Employed | 76 | 36.4% |  |  |
| Unemployed/Other | 35 | 16.7% |  |  |
| Income |  |  | 1.95 | .96 |
| Up to 15.000 Euros | 81 | 38.8% |  |  |
| 15.001 to 28.000 Euros | 75 | 35.9% |  |  |
| 28.001 to 55.000 Euros | 40 | 19.1% |  |  |
| 55.001 to 75.000 Euros | 9 | 4.3% |  |  |
| Over 75.000 Euros | 4 | 1.9% |  |  |
| Political orientation |  |  | 5.14 | 1.12 |
| Extreme right | 0 | 0% |  |  |
| Right | 7 | 3.3% |  |  |
| Moderate right | 16 | 7.7% |  |  |
| Center | 19 | 9.1% |  |  |
| Moderate left | 75 | 35.9% |  |  |
| Left | 83 | 39.7% |  |  |
| Extreme left | 9 | 4.3% |  |  |

*Descriptive of the sample’s characteristics (Study 2; N = 350)*

| **Variable** | ***Frequency*** | ***Percentage*** | ***M*** | ***SD*** |
| --- | --- | --- | --- | --- |
| Education |  |  | 2.85 | .98 |
| Middle school diploma | 8 | 2.3% |  |  |
| High school diploma | 152 | 43.4% |  |  |
| Bachelor’s degree | 93 | 26.6% |  |  |
| Master’s degree | 82 | 23.4% |  |  |
| Postgraduate level | 11 | 3.1% |  |  |
| Other | 4 | 1.1% |  |  |
| Employment |  |  | 1.63 | .69 |
| Student | 170 | 48.6% |  |  |
| Employed | 138 | 39.4% |  |  |
| Unemployed/Other | 42 | 12% |  |  |
| Income |  |  | 1.92 | .95 |
| Up to 15.000 Euros | 149 | 42.6% |  |  |
| 15.001 to 28.000 Euros | 100 | 28.6% |  |  |
| 28.001 to 55.000 Euros | 82 | 23.4% |  |  |
| 55.001 to 75.000 Euros | 17 | 4.9% |  |  |
| Over 75.000 Euros | 2 | 0.6% |  |  |
| Political orientation |  |  | 4.93 | 1.20 |
| Extreme right | 0 | 0% |  |  |
| Right | 14 | 4% |  |  |
| Moderate right | 37 | 10.6% |  |  |
| Center | 56 | 16% |  |  |
| Moderate left | 107 | 30.6% |  |  |
| Left | 123 | 35.1% |  |  |
| Extreme left | 13 | 3.7% |  |  |

*Descriptive of the sample’s characteristics (Study 3; N = 244)*

| **Variable** | ***Frequency*** | ***Percentage*** | ***M*** | ***SD*** |
| --- | --- | --- | --- | --- |
| Education |  |  | 2.78 | .91 |
| Middle school diploma | 4 | 1.6% |  |  |
| High school diploma | 109 | 44.7% |  |  |
| Bachelor’s degree | 77 | 31.6% |  |  |
| Master’s degree | 45 | 18.4% |  |  |
| Postgraduate level | 8 | 3.3% |  |  |
| Other | 1 | 0.4% |  |  |
| Employment |  |  | 3.08 | 2.76 |
| Student | 114 | 46.7% |  |  |
| Employed | 93 | 38.1% |  |  |
| Unemployed/Other | 37 | 15.1% |  |  |
| Income |  |  | 1.90 | .94 |
| Up to 15.000 Euros | 100 | 41% |  |  |
| 15.001 to 28.000 Euros | 83 | 34% |  |  |
| 28.001 to 55.000 Euros | 52 | 21.3% |  |  |
| 55.001 to 75.000 Euros | 3 | 1.2% |  |  |
| Over 75.000 Euros | 6 | 2.5% |  |  |
| Political orientation |  |  | 4.86 | 1.20 |
| Extreme right | 1 | 0.4% |  |  |
| Right | 6 | 2.5% |  |  |
| Moderate right | 30 | 12.3% |  |  |
| Center | 48 | 19.7% |  |  |
| Moderate left | 76 | 31.1% |  |  |
| Left | 71 | 29.1% |  |  |
| Extreme left | 12 | 4.9% |  |  |
